# Remarks on Neuralgia

**Published:** 1872-03

**Authors:** Robert Robson

**Affiliations:** New Harmony, Posey County, Indiana


### REMARKS ON NEURALGIA.

BY ROBERT ROBSON, M. D., NEW HARMONY, POSEY COUNTY, INDIANA.

The following remarks, on the subject of neuralgia, I am aware, presents nothing new to the intelligent physician, and certainly nothing original; they are simply the result of long practice and experience in the profession, combined with that of others. In a former article published in the Medical Examiner of Chicago, I remarked that it, neuralgia had been of frequent occurrence in this locality, and demanded considerable attention at the hands of the practicing physician. That though the doctrine of spinal neuralgia was founded on anatomy and physiology, and that experience and observation, daily adding their sanction to the deduction of science, yet, notwithstanding, there was no subject so much involved in obscurity as that of the true pathology of neuralgia. And unless we take the most comprehensive view of the pathology of this most distressing disease, we will certainly attack the shadow, and not the substance of the insideous enemy with which we are contending, and especially if we direct our chief efforts to those points in which its energies appear to be expended.

It is the impression of very many physicians, and I think there is no doubt of it, that disease of the viscera affecting the trunk of a nerve, gives rise to pain and suffering, which is felt at the extremity of its filiments, and that all the branches proceeding from such trunk, will have a similar disturbance of function from the same morbid impression, and that the affected nerves resolve themselves into those of excitement, or defective energy, each having its own peculiar forms of disease,—those of excitement, as for instance, neuralgia, most probably the result of pressure, mechanical impulses, or vascular congestion or inflammation, etc., while that of diminished energy, probably the result of atrophy gives rise to adynamic disease, etc. That the seat of pain is not the seat of disease, in neuralgia, is undoubtedly correct, the only exception that can be made is, where cutaneous nerves are injured, then the integument of the part suffer pain. There are many points which go to prove the sympathy existing between the viscera and nervous system, such as the pain and numbness in the shoulder from chronic hepatitis, the headache from disordered stomach or liver, the numbness of the lower extremities from the calculus of the kidneys; also, that wherever the median or radial nerve becomes affected by pressure, the finger and thumb, which it supplies, lose their sensibily, and in the same way, when the sciatic becomes influenced, the foot will be paralyzed; we have, also, the severe pains in the head, back, and extremities as in our bilious fevers, and other disorders, accompanied with marked derangement of the secretions of the viscera, etc. The form of neuralgia, generally prevalent in the Wabash bottoms, I have ever regarded, for many years, as a disguised intermittent, having the same origin with cur bilious fever, and have treated it, for many years with general success, with mercurial cathartics, quinine and iron as the best remedial agents, to which it generally yields. My own experience of upward of forty years, in the same locality, is, that much the largest portion of neuralgic cases, that come under notice, are the result of derangement of some of the internal viscera. I will endeavor to illustrate, by a few interesting cases, out of many that have come under my observation in this section. I feel that the before-mentioned principles, which, if acknowledged by some practitioners, and adopted, would conduce very much to the mitigation of human suffering.

Case 1. Some three or four years since, I was called to visit Mrs. H——, living three miles from New Harmony. On my arrival, I found her suffering the most excruciating pains in the face, the side of her head and neck, and at all times attended with a constant grumbling, though acutely paroxysmal in its general character. There was no fever, tumefaction or heat, it was a clear case of neuralgia, from which she had not been free for many months, indeed, such was her suffering, that she seemed on the verge of the grave. She told me that doctors had done all that could be done for her, she believed—she had been bled, blistered, and taken medicine for months without any permanent relief. Under such circumstances, I strongly suspected local irritation of the nerves, or disease of the alveolar contiguous to it, and carefully examined her teeth, and proceeded to extract one very much decayed, and while doing so, accidentally tapped, with the forceps, those immediately adjacent, which caused the most intense suffering to the patient, the tooth extracted was connected, at the point of the fang, with an exostosis as large as a small pea. I then advised, and with her permission, continued to extract six more, all of which were analagous to the first, having more or less osseous deposit on the fang; after which, she was very much relieved, and after the use of gentle stimulants and tonics, she gained strength and became comparatively well, and died about three years afterward. This I consider a clear case of neuralgia, mainly if not entirely attributable to the osseous pressure and irritation of the second or third branch of the fifth pair of nerves, which was cured by removing the cause.

Case 2. Some time since, I was called to a lady, one of my neighbors, who was seized with a most violent pain in the suborbital region of the left side of the face, preceded by vomiting and shiverings; the pulse, during the paroxysm of pain, was small, the skin hot and dry ; the tongue white and furred : she was extemely restless, with a sensation of general uneasiness, which diminished during the night, when the pain of the face subsided. In the morning the pain would return, with apparently renewed violence, and thus had continued for some days before I saw her. During this time, she had taken no medicine, when I prescribed a dose of calomel and opium, followed by a mild aperient, after which, she took liberal doses of quinine, precip. carbonate of iron, ext. taraxacum and brandy, after which, she soon recovered.

Case 3. It is now upward of thirty years, since I was first called to visit a lady, of this place, possessing a good natural constitution, of a nervo-bilious temperament, who is now upward of seventy years of age, who has suffered more or less, almost every year during that time, with a severe spasmodic form of hysteria, occasionally alternating with neuralgia. During her earlier life, her attacks were mainly hysterical, and was treated accordingly. Latterly, and for several years, they have been neuralgic, gradually assuming a more violent form as she advances in years, affecting the spinal column, and intercostal and abdominal muscles,—in short, sometimes the system seems involved, you cannot touch her, without inflicting intense pain. Should she desire to change her position during the intensity of the paroxysm, the gentlest assistance of the nurse causes so much suffering, that her screams can be heard all over the house; her pulse was small, her skin hot and dry, her tongue furred, and especially toward the root. At that time, I regarded her case as one consequent on disease of the spinal cord, and treated it with hot anodyne fomentations, afterward with blisters to the spinal column, with a strong liniment of the extract of atropia belladonna, dissolved in pure acetic acid, well-rubbed into other parts affected, with sulph. morph. internally, attention to the bowels, etc., but without any relief of her sufferings. I now began to feel that I was mistaken in the pathology of the case, that the cause was probably remote from the seat of pain; that the case was one consequent on visceral derangement, and administered calomel, in purgative doses, followed by aperients, which produced copious dark fœtid bilious passages, with excellent effect, when after a few doses of stimulants and tonics, she rapidly recovered. In the month of March last, I again attended this lady in one of the most violent attacks of neuralgia I ever witnessed, which continued to harrass the patient, notwithstanding my efforts to the contrary. Mercurials, opiates, stimulants and tonics, with various auxilliaries all failed in this case, when it occurred to me to try the hydrate of chloral, when I dissolved hydrate of chloral, dr. ss., in aqua cinnamon, oz. i., one-third of which I gave every hour, when, to the surprise of all present, her suffering abated, and she fell into a doze, and finally into sound sleep, and slept several hours, and awoke up very much relieved, and in a few days enjoyed her usual health.

CASE 4. Some few weeks since, I was in attendance on a lady in New Harmony, who, during the turn of life, was suffering much with visceral derangement, and especially with severe aching pains in the liver, who had taken a regular course of alteratives, followed by aperients, etc., under which treatment she was rapidly improving, when, to my surprise, I was again sent for, and found her suffering the most acute pains in the eye-balls and suborbital regions of both sides of the face, her eyes were much inflamed—light obnoxious—her pulse small and weak, and her skin below the natural temperature, tongue clean. The treatment consisted of warm anodyne fomentations to the eye-balls, with quinine and per. sulph. of iron, internally; attention to the bowels, etc., which was continued for several days, when she became extremely restless, when thirty grains of the hydrate of chloral was given in broken doses, the patient now enjoyed five hours of sound sleep, after which, she recovered. Observation and experience are the only true foundation of real knowledge in medicine, as in every other branch of physical science, and the pratice based upon it, will inevitably be most successful, and until we find the key to unlock the nervous system, and give us more light on many of its obscure diseases, it is wisdom to pursue the course most generally attended with success. It has long been my impression that every form of neuralgia, except those arising from pressure on the trunk of the nerve, from exostosis and other bony structures, and those resulting from blows and other mechanical injuries, are the result of visceral derangement, the morbid action of which, being communicated through the spinal branches of the great sympathetic, to the spinal marrow and trunks, gives rise to that exaltation of nervous sensibility and morbid impressibility which exist in so high a degree in this truly painful disease.

